# Dendritic Cell-Based Approaches for Therapeutic Immune Regulation in Solid-Organ Transplantation

**DOI:** 10.1155/2013/761429

**Published:** 2013-10-24

**Authors:** Giuseppe Vassalli

**Affiliations:** ^1^Department of Cardiology, Centre Hospitalier Universitaire Vaudois, 1011 Lausanne, Switzerland; ^2^Molecular Cardiology Laboratory, Fondazione Cardiocentro Ticino, 6900 Lugano, Switzerland

## Abstract

To avoid immune rejection, allograft recipients require drug-based immunosuppression, which has significant toxicity. An emerging approach is adoptive transfer of immunoregulatory cells. While mature dendritic cells (DCs) present donor antigen to the immune system, triggering rejection, regulatory DCs interact with regulatory T cells to promote immune tolerance. Intravenous injection of immature DCs of either donor or host origin at the time of transplantation have prolonged allograft survival in solid-organ transplant models. DCs can be treated with pharmacological agents before injection, which may attenuate their maturation *in vivo*. Recent data suggest that injected immunosuppressive DCs may inhibit allograft rejection, not by themselves, but through conventional DCs of the host. Genetically engineered DCs have also been tested. Two clinical trials in type-1 diabetes and rheumatoid arthritis have been carried out, and other trials, including one trial in kidney transplantation, are in progress or are imminent.

## 1. Introduction

Allogeneic cell and solid-organ grafts trigger immune responses that cause destructive graft rejection in the absence of sustained drug-based immunosuppression [1]. Unfortunately, the latter is associated with major side effects including severe infections and cancer, especially after many years of treatment. Moreover, immunosuppressive drugs have failed to prevent chronic graft rejection, which is primarily manifested by allograft vasculopathy. The hope in the immunotherapy field is to develop a therapy that targets and neutralizes the alloimmune response selectively, while leaving protective immunity intact.

Patients with a functioning allograft in the absence of maintenance immunosuppression have been described occasionally [2]. In some of them, immunosuppressive drugs were discontinued because of infection or cancer [3]. Others decided to stop taking the drugs [4]. Some liver transplant recipients were enrolled in studies of weaning immunosuppression [5]. Moreover, some kidney transplant recipients participated in studies of tolerance induction [6–9] which, in part, included hematopoietic cell transplantation [8, 9]. These reports demonstrate the sporadic occurrence of operational tolerance, as defined by lack of destructive graft rejection in the absence of maintenance immunosuppression [10].

Sixty years ago, Billingham et al. described “actively acquired tolerance” of foreign cells. They claimed that, “if the first presentation of foreign cells takes place in fetal life, resistance to a graft [from the same donor or from some other member of the donor's strain] transplanted on some later occasion is abolished or at least reduced” [11]. This concept implied the existence of regulatory mechanisms, other than deletion of donor-reactive immune cells, favoring graft acceptance. Thirty years later, Hall et al. reported that rats that accepted cardiac allografts for extended periods of time in the absence of treatment with cyclosporine or other immunosuppressive drugs had suppressor T cells that actively prevented graft rejection [12].

Multiple leukocyte populations including dendritic cells (DCs), macrophages, T and B cells, and natural killer (NK) cells participate in the immune reaction against the allograft [1]. However, these populations include subsets of cells showing regulatory activity. Analyses of immune cells in patients who did not reject the allograft in the absence of drug-based immunosuppression revealed major roles for regulatory T cells (Tregs) [13–15], regulatory B cells [16–18], and regulatory DCs in the establishment of operational tolerance. Regulatory immune cells are selected to either exert inhibitory functions during development or acquire regulatory activity within the graft or in draining lymphoid tissue [2]. The balance between donor-reactive effector cells and regulatory cells in the graft and its environment is naturally shifted towards effector cells and graft rejection. In this review, we wish to summarize our current understanding of tolerogenic DCs favoring the development of antigen-specific immune tolerance and graft acceptance (Table 1). Emerging DC-based approaches for therapeutic immune modulation in solid-organ transplantation, with a focus on heart transplantation, are briefly discussed.

## 2. DC Populations

**Figure 1:**
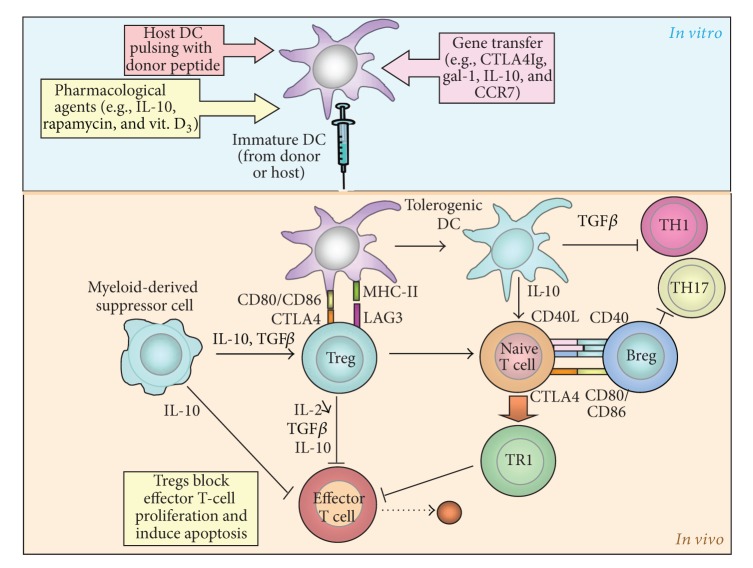
Regulatory T cells (Tregs) are present in the host at the time of transplantation and are recruited to the allograft. They respond to donor alloantigen through cross-reactivity and inhibit T-cell proliferation in draining lymphoid tissue. Tolerogenic DCs favor the generation of Tregs from naive T cells, which then block effector T-cell proliferation while also triggering apoptosis of these cells. They inhibit TH1 cells. These processes facilitate allograft acceptance through several mechanisms including the production and release of immunosuppressive cytokines, such as IL-10 and TGF*β* (modified from Wood et al. [2]).

DC and monocyte lineages originate from a common monocyte and DC progenitor (MDP) that gives rise to monocytes and committed DC progenitors (CDPs) in the bone marrow. CDPs then give rise to pre-DCs that migrate to lymphoid and nonlymphoid tissue and differentiate into the two major populations of lymphoid tissue DCs and nonlymphoid tissue CD103^+^ DCs [22, 23, 40]. Acting as antigen-presenting cells, DCs trigger innate and adaptive immune responses to microbial antigens and alloantigens, as well as to self-antigens in autoimmune diseases. DCs initiate the innate immune response through activation of NK cells. They also set off the adaptive immune response through activation of naive B and T lymphocytes. Besides their traditional role as immunogenic cells, DCs regulate the immune reaction and mediate peripheral T-cell unresponsiveness under homeostatic conditions *in vivo* [41]. The regulatory activity of DCs can be accounted for by multiple mechanisms including the production and release of anti-inflammatory cytokines [32], Fas/Fas-ligand-induced apoptosis of effector immune cells [42], deletion, and induction of Tregs [39, 43–46] (Figure 1). Tolerogenic DCs comprise a majority of immature DCs and subpopulations of DCs with various maturation stages, including plasmacytoid DCs [43]. However, a major drawback *in vivo* is the potential of tolerogenic DCs to mature during infections or inflammation, which would convert them into immunogenic cells [47].

DCs are a heterogeneous population. At least seven DC subpopulations have been defined in mice based on differential expression of surface and intracellular markers [48–53]. The relationships between mouse and human DC populations are incompletely understood; however, recent comparative genomics studies have revealed functional equivalences between DC subsets in the two species [25]. Based on morphology, cell-surface markers, and gene expression profiles, DCs have traditionally been subdivided into classical and plasmacytoid DCs [24, 27, 54–57]. The latter are characterized by the CD11c^low^CD45RA^hi^MHC-II^low^ immunophenotype in mice and the CD4^hi^CD8^neg^CD11c^low^CD45RA^hi^MHC-II^low^ immunophenotype in human [2].

Alternatively, a development-oriented nomenclature has also been used [23]. This classification subdivides DCs into conventional and monocyte-derived DCs. It is based on the assumption that monocytes give rise to mucosal DCs, but not to splenic, conventional DCs [58]. The existence of genuine monocyte-derived DCs *in vivo* is presently being explored. At steady state, most DCs in mouse lymphoid organs do not arise from monocytes and depend upon fms-like transcript 3 ligand (Flt3L) for their development [59]. However, some tissue (e.g., the gut) harbor macrophage colony-stimulating factor (M-CSF)—dependent monocytes at steady state [60, 61]. Langerhans cells are DCs within the epidermis and other squamous epithelia, which rely on M-CSF for their development [60, 62]. In histiocytosis X, the expansion of Langerhans cells is manifested by either granuloma formation or a more diffuse proliferative disease [63].

Another classification subdivides DCs into resident and migratory DCs [23]. Resident DCs traffic directly to lymphoid tissue from a blood precursor, whereas migratory DCs first enter tissue before moving to lymph nodes [64–66]. DC populations express distinct pattern recognition receptors [67] and play different roles in nature. Monocyte-derived DCs can be observed in lymphoid tissue in response to infection with *Leishmania major* [68] or *Listeria monocytogenes* [69].

The development of DCs with either immunogenic or regulatory activity depends on a series of biological processes including differentiation, expansion, migration, antigen uptake and processing, and maturation.

## 3. DC Maturation

Under homeostatic conditions, tissue-resident DCs are immature and express low levels of major histocompatibility complex (MHC) class II and little or no T-cell costimulatory molecules. The role of immature DCs is not precisely defined, as they can give rise to either mature DCs or semimature regulatory DCs that share some phenotypic traits of mature DCs (e.g., CCR7 chemokine receptor expression [70]). Immature DCs sense the presence of pathogen- and damage-associated molecular patterns (PAMPs and DAMPs) from microbial agents and damaged cells, respectively, and activate proinflammatory molecular signaling cascades, such as nuclear factor kappa B (NF*κ*B) and interferon responsive factor (IRF) pathways [71, 72]. Activation of these signaling pathways is associated with DC maturation. Maturing DCs lose their ability to take up soluble antigen and acquire T-cell stimulatory activity as a result of increased antigen processing capacity and upregulation of MHC, T-cell costimulatory molecules, and proinflammatory cytokines [73, 74]. Traffic molecules (e.g., CCR7) are upregulated in maturing DCs, allowing them to migrate to lymphoid tissue [70, 75–78]. *In vitro*, mature DCs present “probing movements” characterized by the incessant lengthening and retraction of long processes from the cell body [79]. Steady-state DCs in intact lymph nodes *in vivo* extend and retract processes in a fairly similar way [80]. Immature DCs and, to a lesser extent, mature DCs take up particles and apoptotic cells [81, 82]. Uptake of apoptotic cells has been implicated in antigen cross-presentation, a mechanism that entails either immune tolerance under homeostatic conditions or immune activation upon DC maturation [83]. In addition, DCs participate in two “nonclassical” pathways of antigen presentation that involve autophagy of cytosolic components for presentation on MHC class II and cross-presentation of endocytosed substrates on MHC class I [84]. Peptide-MHC-class II complexes formed in lysosomes during DC maturation move to the cell surface [85–87] and interact with the T-cell receptor. Maturing DCs upregulate T-cell costimulatory molecules including CD80, CD86, CD40, OX40L, and inducible T-cell costimulator ligand (ICOSL/CD275) [88, 89], along with proinflammatory cytokines (e.g., IL-1*β*, IL-2, IL-6, IL-8, IL-12, and IL-18).

The costimulation paradigm dictates that strong T-cell activation requires three distinct signals: the antigenic stimulus provided by MHC presenting a cognate peptide, costimulatory molecules, and stimulatory cytokines. Antigen presentation in the absence of strong costimulation can induce a state of antigen-specific T-cell unresponsiveness or anergy. Repetitive stimulation with allogeneic immature human DCs has been shown to induce IL-10-producing, nonproliferating CD4^+^ T cells with regulatory activity [90]. Systemic injection of immature, but not of mature, donor bone marrow-derived CD8*α* ^−^ DCs to the recipient before engraftment prolonged cardiac allograft survival in the absence of immunosuppression in mice [91, 92]. Both immature and comparatively mature CD8*α* ^+^ “lymphoid-related” DCs injected 7 days before transplantation likewise prolonged cardiac allograft survival in fully MHC-mismatched mice [93]. Moreover, systemically injected donor DCs prevented rejection of MHC-mismatched skin grafts [94] and lethal acute graft-versus-host disease [95] in mice. Immature DCs derived from recipient bone marrow progenitors similarly improved cardiac allograft survival in mice [96].

Immature DCs with inhibitory properties can be generated from monocyte precursors *in vitro* [97]. Alternatively, immature DC precursors can be mobilized in the live donor by Flt3L or granulocyte-colony stimulating factor (G-CSF), collected by leukapheresis, purified, and injected to the recipient before transplantation. However, this is not a feasible method with deceased donors, such as in heart transplantation. In human, bone marrow cell mobilization by G-CSF preferentially increased circulating plasmacytoid DC precursors [98].

## 4. DCs and Tregs

DCs continuously present harmless self- and nonself-antigens in a way that favors tolerance. Following antigen presentation in the absence of costimulatory signals in mice, immature DCs induce conventional naive T cells to convert into Tregs and boost the regulatory activity of existing Tregs at once [99–106]. Immature DCs in secondary lymphoid tissue accumulate antigen and bolster the generation of Tregs that promote antigen-specific tolerance. Mice lacking functional immature DCs develop fatal autoimmune disease, possibly owing to low numbers of Tregs [19, 61, 107, 108]. Mammals, including human, lacking functional Tregs likewise develop fatal autoimmune disease [109].

Regulatory DCs interact with Tregs in bidirectional ways. Tregs express inhibitory receptors, such as cytotoxic T-lymphocyte antigen 4 (CTLA4), lymphocyte-activation gene 3 (LAG-3), and glucocorticoid-induced tumor necrosis factor receptor (GITR), along with anti-inflammatory cytokines such as TGF-*β*, IL-10, and IL-35 [110, 111]. TGF-*β* is a central mediator in the generation of Tregs [112, 113]. These cells can be subdivided into CD4^+^ natural Tregs that are formed during thymic development in the fetus [114–116] and adaptive Tregs. The transcription factor Foxp3 is upregulated in developing T cells upon recognition of self-antigen in the thymus and controls the function of Tregs [114, 117]. Natural Tregs can convert into adaptive CD4^+^ or CD8^+^ Tregs in secondary lymphoid organs. In two human volunteers, a single subcutaneous injection with autologous immature monocyte-derived DCs pulsed with influenza matrix peptide induced IL-10-producing, peptide-specific CD8^+^ Tregs and suppressed peptide-specific killing activities of CD8^+^ T cells [118]. These results indicate that immature DCs can induce Tregs and inhibit antigen-specific effector T cells in human.

## 5. Tolerogenic DCs

Immature DCs are comprised of distinct populations that differ in their capacity to present antigen, produce cytokines, and promote tolerance. Phenotypically mature DCs are not always immunogenic. For instance, immature DCs that upregulate MHC and costimulatory molecules after *in vitro* treatment with TNF-*α* or IFN-*γ*, a typical attribute of mature DCs, but are still able to promote the generation of Tregs from naive T cells, have been described [119, 120]. Also, some immature DCs in peripheral tissue express high levels of CCR7, another typical feature of mature DCs, while maintaining the capacity of inducing Tregs [121–124]. CCR7 deficiency prevents lymphatic migration of immature DCs and induction of inhaled and oral tolerance, indicating a major role for this chemokine receptor in tolerance induction [125].

Plasmacytoid DCs, originally identified as a subset of DCs that produce type I interferons in response to viral infection, differ from traditional DCs in several ways including decreased costimulatory molecule expression and reduced allostimulatory capacity. Human plasmacytoid DCs preferentially express immunoglobulin-like transcript 7 (ILT7) that couples with a signaling adapter to activate a prominent immunoreceptor tyrosine-based activation motif- (ITAM-) mediated signaling pathway [27]. Nonlymphoid, tissue-resident plasmacytoid DCs are key cellular players in the regulation of mucosal immunity at steady state and in the induction of central and peripheral tolerance. They have been associated with generation of alloantigen-specific CD4^+^ Tregs or CD8^+^ Tregs that promote graft tolerance [26, 54, 57, 126–133] and long-term cardiac allograft survival [131, 132].

Natural tolerogenic DCs originate from hematopoietic precursors and maintain tolerance constitutively under homeostatic conditions [134]. In the thymus, where central tolerance is set up, natural tolerogenic DCs induce the generation of Foxp3^+^ natural Tregs by a mechanism that is mediated by IL-7-related thymic stromal lymphopoietin (TSLP) produced by Hassall's corpuscles in the thymic medulla [135]. Induced tolerogenic DCs differ from natural DCs in that their tolerogenic activity is controlled by molecular signals from the environment, such as TGF-*β* and IL-10 in the intestinal mucosa, which oppose maturation stimuli. *In vitro* treatment of immature DCs with these anti-inflammatory cytokines promotes the development of tolerogenic DCs that assist in the conversion of naive T cells into Tregs more effectively than the immature DCs themselves. Several strategies have been developed to generate tolerogenic DCs for therapeutic use in organ transplantation [44, 136–142]. The two major general approaches consist of pharmacological conditioning and genetic engineering of immature DCs.

### 5.1. Pharmacologically Conditioned DCs

The development of immunosuppressive drugs has traditionally been focused on inhibition of lymphocyte proliferation, which was initially perceived as a key process in immune responses to all kinds of antigens. By screening many agents for inhibition of lymphocyte proliferation, Schwartz and Dameshek discovered the immunosuppressant 6-mercaptopurine in 1959 [143]. Over the past two decades, it has been increasingly recognized that many drugs that primarily act by inhibiting T-cell proliferation concurrently modulate DC function.

Glucocorticoids inhibit DC differentiation and maturation both *in vitro* and *in vivo *[144–150]. Dexamethasone preferentially blocked the differentiation of plasmacytoid DCs and enhanced their apoptotic death [150]. DCs conditioned by vitamin D_3_ *in vitro* exhibited several attributes of tolerogenic DC, such as resistance to maturation, IL-10 release upon stimulation, and low T-cell allostimulatory capacity [151, 152]. Human monocyte-derived, vitamin D_3_-treated DCs displayed a semimature phenotype, anti-inflammatory properties, and low T-cell allostimulatory capacity [153]. T cells from patients with relapsing remitting multiple sclerosis cultured with autologous vitamin D_3_-treated DCs loaded with myelin peptides induced hyporesponsiveness of myelin-reactive T cells [153]. Synergistic effects of vitamin D_3_ and dexamethasone have been reported [149]. Vitamin D_3_ combined with dexamethasone was used to derive tolerogenic DCs from healthy subjects and from patients with rheumatoid arthritis [154]. *In vitro* LPS-stimulated tolerogenic DCs, referred to as “alternatively activated” DCs, exhibited enhanced ability to migrate to lymphoid tissue and to present antigen. They induced memory T-cell hyporesponsiveness, proliferation of naive T cells, low IFN-*γ* expression, and high IL-10 expression [155, 156].

DCs conditioned by IL-10 or other immunosuppressive cytokines *in vitro* promoted antigen-specific anergy and regulatory activity in memory CD4^+^ T cells [21, 157]. Two different populations of DCs were derived from monocytes cultured in the presence of GM-CSF and IL-4, depending on the time point of their exposure to IL-10. When IL-10 was added at the end of culture, DCs showed an immature phenotype, were resistant to maturation [158, 159], and induced antigen-specific CD4^+^ and CD8^+^ T-cell anergy [159–161]. DCs derived from macaque monocytes treated with vitamin D_3_ and IL-10 were resistant to maturation and displayed low T-cell allostimulatory activity *in vitro* [162]. Systemic injection of these DCs to MHC-mismatched recipient macaques treated with antihistamine drug and CTLA4Ig (soluble CTLA4-immunoglobulin fusion protein) elicited a transient increase in donor antigen-specific T-cell proliferation, with no changes in antidonor antibodies [162]. Conversely, when IL-10 was added at the initiation of monocyte culture, the differentiating DCs expressed CD83, CD80, and CD86, similar to mature cells, but also Ig-like transcript- (ILT-) 2, ILT3, ILT4, and human leukocyte antigen G, similar to tolerogenic DCs [163]. Importantly, IL-10-treated DCs supported the differentiation of type-1 Tregs (Tr1) [163, 164]. On the other hand, IL-10 downregulated CCR7, thereby impairing lymphoid homing of DCs *in vivo* [165, 166].

Cyclosporine A and tacrolimus (FK-506), two calcineurin inhibitors, inhibited IFN-*γ* production and MHC-restricted antigen presentation by myeloid DCs [167], as well as activatory interactions of DCs with T cells [168]. In one study [169], cyclosporine A downregulated chemokine receptors and cyclooxygenase-2 (COX-2). This effect was associated with impaired DC migration. However, another study [166] showed that cyclosporine A and tacrolimus, unlike dexamethasone and IL-10, did not downregulate CCR7 nor did they inhibit CCL19 chemokine-mediated migration of human monocyte-derived DCs *in vitro* or lymphoid homing of mouse DCs *in vivo*.

Another important molecule used to generate tolerogenic DCs is mycophenolate mofetil. This agent inhibits T- and B-cell proliferation by interfering with *de novo* synthesis of purines required for the production of nucleic acids. Mycophenolate mofetil inhibits the maturation of DCs *in vitro* and their immunostimulatory capacity *in vivo *[170].

Aspirin [171–174] and deoxyspergualin [175], two inhibitors of the NF-*κ*B signaling pathway, likewise inhibit DC maturation. Immature host DCs conditioned *ex vivo* with oligodeoxynucleotides (ODN) against NF-*κ*B achieved long-term cardiac allograft survival in rats [176]. Blocking NF-*κ*B signaling during human DC differentiation induced anergy and Treg activity for one, but not two, HLA-DR mismatches [177]. Interestingly, a clinical study showed a significant correlation between low-dose aspirin therapy and improved allograft function and survival in kidney transplant recipients [178]. Systemic administration of immature autologous myeloid DCs combined with a deoxyspergualin derivative induced donor-specific tolerance in a heart transplant model in rats [20]. This effect was associated with an increase in NK^−^TCR*αβ* ^+^CD3^+^CD4^−^CD8^−^double negative Tregs, a population that has been linked to donor-specific improvement of graft survival [179], but not in conventional CD4^+^CD25^+^Foxp3^+^ or CD8CD45RC^low^ Tregs in host spleens. The double negative Treg population expressed IFN-*γ*. This cytokine is produced by alloantigen-reactive CD4^+^Foxp3^+^ Tregs and enhances their regulatory activity *in vivo *through an autocrine mechanism [180]. IFN-*γ* also induces the development of Foxp3^+^ Tregs from CD4^+^CD25^−^cells. These findings indicate that IFN-*γ* released by double negative Tregs potentiates their ability to suppress alloreactive CD4^+^ and CD8^+^ T cells [181].

Rapamycin, an immunosuppressive macrolide, inhibits the integrative kinase mammalian target of rapamycin (mTOR). Inhibition of mTOR exerts immunoregulatory activity [182] including attenuated innate immune responses [183]. Mouse monocyte-derived DCs conditioned by rapamycin *in vitro *were resistant to maturation induced by inflammatory stimuli while conserving their ability to migrate to lymphoid tissue and to enrich the naturally occurring CD4^+^ Tregs. Intravenous injection of host rapamycin-treated DCs pulsed with donor antigen before heart transplantation, combined with a short course of immunosuppression, prolonged allograft survival indefinitely in rodents [28–30, 184, 185]. Rapamycin-treated DCs expressed CD83, CD86, low levels of IL-10, and high levels of IL-12p40/p70 [186]. Although these features were typical of a mature phenotype, these DCs inhibited T-cell alloproliferation, similar to those treated with vitamin D3 and dexamethasone [187]. Upon LPS stimulation, they expressed high levels of IL-12 [28], a cytokine that participates in the generation of Foxp3^+^ Tregs. However, human rapamycin-treated DCs, unlike murine, were only partially maturation resistant *in vivo *[30].

DC-mediated NK T-cell activation is a critical event in the early immune response to renal ischemia/reperfusion injury. DCs treated with adenosine 2A receptor (A_2_AR) agonists protected the kidney from ischemia/reperfusion injury through suppression of IFN-*γ* production by NK T-cells and decreased costimulatory molecule expression [31]. In a recent study [188], protein kinase C inhibitors induced the development of stable human tolerogenic DCs.

### 5.2. DCs Generated in the Presence of Low-Dose GM-CSF without IL-4

A combination of high-dose GM-CSF and IL-4 has been used as a standard protocol for generation of DCs from bone marrow precursors *in vitro*. Although DCs generated using this protocol induced primary allogeneic T-cell anergy *in vitro*, they were not resistant to maturation. The potential of tolerogenic DCs to mature within an inflammatory environment, turning into immunogenic cells *in vivo*, is of major concern to therapeutic applications [47]. An alternative protocol consists of culturing bone marrow precursors with low-dose GM-CSF in the absence of IL-4. Mouse DCs generated using this protocol efficiently captured and presented antigen, were maturation-resistant and poor stimulators of T-cell proliferation, and prolonged cardiac allograft survival *in vivo* [92]. DCs derived from monkey bone marrow in the presence of GM-CSF without IL-4 gave rise to two populations showing a similar phenotype but different functional properties. The adherent population displayed immunoregulatory properties that were correlated with upregulation of heme oxygenase-1 (HO-1), an anti-inflammatory enzyme, both *in vitro* and in a rat allotransplant model *in vivo*. Conversely, the nonadherent population was immunogenic [189]. Immature DCs derived from human bone marrow by low doses of GM-CSF in the absence of IL-4 were resistant to common maturation stimuli and exhibited tolerogenic properties *in vitro* [47, 190], including induction of T-cell anergy in naive allogeneic T cells when stimulated twice with immature DCs [47]. Therefore, the establishment of the anergic state appeared to require two subsequent stimulations by immature DCs.

### 5.3. Tolerogenic DC Therapy Combined with Immunosuppressive Drug Administration

*In vivo *injection of immature DCs can be combined with the systemic administration of immunosuppressive drugs. For instance, immature donor DC therapy combined with either anti-CD154 (CD40 ligand) antibody [191] or anti-CD54 (intercellular adhesion molecule) antibody together with CTLA4Ig [192] prolonged cardiac allograft survival in mice for more than 100 days. Likewise, adoptive transfer of immature donor DCs combined with anti-CD154 antibody inhibited intimal hyperplasia and arterial lesions in mouse aortic allografts [193]. Alloantigen-pulsed, rapamycin-treated DCs combined with a low dose of rapamycin administered at the time of transplantation induced indefinite cardiac allograft survival in mice [184]. Moreover, mature donor DCs combined with a systemic treatment with tacrolimus prolonged cardiac allograft survival in rats, whereas DCs alone were ineffective in this model [194]. Other studies did not support synergistic effects of tolerogenic DC therapy and systemic treatment with rapamycin or cyclosporine A in transplant models [167]. On the other hand, immunosuppressive drugs can modify DC function and hinder the efficacy of tolerogenic DC therapy. As an example, tacrolimus inhibits the ability of DCs to process and/or present antigen [167, 195]. For these reasons, interactions of systemic drug treatments with injected tolerogenic DCs need to be investigated in animal models before the initiation of clinical trials, as discussed below.

### 5.4. Genetically Modified DCs

One critical aspect of tolerogenic DC therapy is the stability of tolerogenic DCs, which should not turn into immunogenic DCs *in vivo *when exposed to proinflammatory cytokines. This consideration is important in transplantation, a condition associated with chronic inflammation. Semimature tolerogenic DCs have been shown to convert into immunogenic DCs in an inflammatory environment *in vivo* [196, 197], which can accelerate graft rejection. Because the conditioning effect by pharmacological agents on DCs *in vitro* may not last *in vivo*, genetic engineering of DCs to express an immunosuppressive factor may represent an attractive approach. Genetically modified DCs can express the therapeutic gene for extended periods of time *in vivo*. Because DCs are manipulated genetically *in vitro*, the recipient is not directly exposed to the gene transfer vector. Recombinant adenovirus vectors have been used in many studies due to their high efficiency, despite their proinflammatory effects that entail DC maturation [198]. Recombinant retrovirus vectors and nonviral gene transfer systems may induce lower levels of DC maturation [199]. With respect to the transgene which is to be constitutively expressed by DCs, candidates include genes that encode an immunosuppressive cytokine, a costimulatory inhibitor, a death ligand that causes killing of effector T cells, a chemokine receptor, or an adhesion molecule that mediates lymphoid homing, among others.

Early studies focused on DCs constitutively expressing immunosuppressive cytokines, particularly IL-10, IL-4, and TGF-*β* [200–208]. These approaches had limited effects on allograft survival. We have shown that bone marrow-derived mouse DCs constitutively expressing indoleamine 2,3-dioxygenase (IDO), an inhibitor of T-cell activation, attenuated T-cell alloproliferative responses in the mixed leukocyte reaction *in vitro *[209]. DCs expressing soluble CTLA4Ig significantly prolonged both cardiac and islet allograft survival in rodents [210–214]. However, this approach was not significantly more effective than CTLA4Ig peptide treatment or direct CTLA4Ig gene delivery into the graft.

By binding the cell death receptor Fas (CD95), Fas ligand participates in the maintenance of peripheral T-cell tolerance and maintenance of immune privilege in certain organs. DCs expressing transgenic Fas ligand inhibited antigen-specific T-cell responsiveness, prolonging the survival of fully MHC-mismatched cardiac allografts in some studies [215, 216], but not in others [217, 218]. Mouse tolerogenic DCs generated with antisense ODN against the costimulatory molecules CD40, CD80, and CD86 remained costimulatory deficient *in vivo*, even after 3 weeks of injection [219]. Low CD40 expression translates into low levels of cytokine (IL-12, TNF-*α*) production upon CD40 ligation [219]. DCs generated with antisense ODN against CD80/86 prolonged allograft survival in a transplant model [220]. Similar findings were reported with immature DCs constitutively expressing soluble TNF-*α* type-1 receptor (sTNFR-1) [221]. DCs engineered genetically to express CCR7 showed enhanced lymphoid homing. Injection of DCs constitutively expressing both IL-10 and CCR7, but not those expressing either gene alone, prolonged cardiac allograft survival in mice [222]. This finding exemplifies synergistic effects resulting from simultaneous targeting of multiple parameters of DC function, such as maturation and lymphoid homing.

Galectin- (gal-) 1 is an endogenous inhibitor of T-cell activation, DC function, and immune cell trafficking [223–225]. DCs deliver tolerogenic signals to T cells through a gal-1-driven immunoregulatory circuit that involves IL-27 and IL-10 [226]. Lung cancer-derived gal-1 mediates DC anergy through inhibitor of DNA binding 3/IL-10 signaling pathway [227]. Gal-1 and -3 genes silencing in immature and mature DCs enhance T-cell activation and IFN-*γ* production [228]. DCs constitutively expressing gal-1 delayed onset of autoimmune diabetes in mice [223]. Moreover, gal-1 prolonged liver allograft survival from Flt3L-pretreated donors in mice [229]. Mice deficient in gal-1 showed accelerated CD8^+^ T-cell-mediated rejection of skin allografts [230]. These findings establish gal-1 as an attractive candidate for gene therapy for transplantation.

### 5.5. DCs Conditioned by Donor Apoptotic Cells and Exosomes

An emerging strategy consists of *in situ* delivery of donor antigen to quiescent host DCs using donor apoptotic cells or exosomes [231, 232]. The latter are 40–100 nm size membrane vesicles rich in microRNA, which play important roles in intercellular communication [233], including the transfer of functional microRNA molecules between DCs [35]. Interaction or phagocytosis of cells in early apoptosis exerts a potent anti-inflammatory and immunosuppressive stimulus on DCs and macrophages. *In situ*-targeting of DCs with donor apoptotic cells restrained indirect allorecognition and attenuated graft vasculopathy in a transplant model [234]. Systemically delivered exosomes rich in donor MHC molecules were taken up by host DCs and prolonged allograft survival [231]. Exosomes from immature DCs combined with rapamycin induced tolerance to cardiac allografts in mice [36].

### 5.6. Tolerogenic DC Therapy May Act through Conventional DCs of the Host

Recent evidence suggests that *in vivo* injected immunosuppressive DCs may attenuate alloresponses and improve cardiac allograft survival, not by themselves, but through quiescent conventional DCs of the recipient [33, 34]. Indeed, transient depletion of conventional DCs of the recipient by the time of DC therapy abolished its beneficial effect on graft survival. This phenomenon was also observed when immunosuppressive DCs were combined with low-dose pharmacological immunosuppression. Following DC therapy, presentation of donor-derived peptides by conventional DCs led to preferential deletion of indirect-pathway CD4^+^ effector T cells, increasing the percentage of Foxp3^+^ CD4^+^ T cells [33]. These results are in line with earlier studies showing that i.v. injection of donor immunosuppressive DCs before heart transplantation inhibited antidonor responses through the direct pathway, which involves recognition of intact donor MHC molecules by recipient T cells [92, 210]. The high precursor frequency of direct-pathway T cells and their predominance in most *in vitro* assays of alloreactivity led to the assumption that the direct pathway was the dominant mechanism of allorecognition during acute cardiac allograft rejection and that systemic injection of donor immunosuppressive DCs inhibited direct responses by interacting with direct-pathway T cells [34]. The aforementioned findings regarding transient depletion of recipient DCs at the time of donor DC therapy [33] demonstrate that depletion of conventional DCs prevents not only downregulation of the indirect T-cell pathway, as anticipated, but also the direct-pathway response elicited by cardiac allografts. This observation suggests that, *in vivo*, donor-derived immunosuppressive DCs by themselves are unable to downregulate direct T-cell responses in the absence of conventional DCs of the recipient [34].

Because generation of donor immunosuppressive DCs is not applicable to deceased donors, use of host DCs pulsed with donor antigen has been viewed as a more feasible method in clinical heart transplantation. Within this context, pulsing host DCs with donor antigen has been used to inhibit the indirect pathway of allorecognition [235, 236]. However, the aforementioned study [33] indicated that systemically injected host immunosuppressive DCs pulsed with MHC class I- or MHC class II-restricted peptides mediated their beneficial effect through conventional DCs of the recipient. These findings suggest that the intrinsic immune regulatory activity of delivered DCs *in vivo* may be less important than what was previously thought. They also raise the question as to whether the function of conventional DCs of the recipients, which are required for the efficacy of DC therapies, is preserved in transplant candidates with an end-stage disease.

### 5.7. Clinical Applications of Tolerogenic DCs

While several clinical trials of immunogenic DC therapy for cancer have been performed [237, 238], clinical DC-based “negative vaccination” is still very much in its infancy. In 2001, a pioneering study showed that autologous immature DCs injected subcutaneously into healthy volunteers were well tolerated, did not cause autoimmunity, and induced antigen-specific CD8^+^ Tregs along with antigen-specific T-cell hyporesponsiveness [118, 239]. Recently, proof of safety for tolerogenic DC therapy was provided by a clinical phase-1 trial in 10 patients with type-1 diabetes who received an intradermal injection of autologous DCs generated in the presence of GM-CSF and IL-4, with or without antisense ODN against CD40, CD80, and C86 [37]. No adverse effects were observed. Meanwhile, two clinical trials of tolerogenic DC therapy in patients with rheumatoid arthritis have been started [38, 240]. Other trials are in progress or are imminent. In transplantation, the OneStudy phase-1 clinical trial has been designed to evaluate tolerogenic DC therapy in renal transplant recipients [39, 241]. Autologous monocyte-derived tolerogenic DCs will be generated from patients with chronic renal failure. Each disease condition for which DC therapy is envisaged can potentially modify the function and *in vivo* survival of tolerogenic DCs derived from the recipient. It therefore is important to test DCs from patients in animal models before starting a clinical trial. Ahead of the clinical trial in kidney transplantation, tolerogenic DCs from patients with chronic renal failure have been validated in a skin transplant model [241]. Moreover, tolerogenic DCs from patients with rheumatoid arthritis were compared with those from healthy controls before the initiation of a clinical trial for this disease; tolerogenic DCs from the two groups showed a similar phenotype and *in vitro *function [154]. Ahead of a clinical trial in patients with relapsing-remitting multiple sclerosis, tolerogenic DCs generated with vitamin D_3_ from these patients were compared with those derived from healthy controls and found to have a comparable phenotype and function [153].

In renal transplant recipients enrolled in the OneStudy trial and treated with tolerogenic DC therapy, drug-based immunosuppression consisting of low doses of mycophenolate mofetil, tacrolimus, and prednisolone is not discontinued [241]. This raises the question as to whether these drugs could modify the function of injected tolerogenic DCs *in vivo*. As already mentioned, immunosuppressive drugs can either enhance or hamper the effect of DC therapy. In preliminary experiments performed ahead of the OneStudy trial, tolerogenic DC therapy did not impair, but actually slightly enhanced, the effect of mycophenolate mofetil on graft survival in a transplant model [241]. While tacrolimus inhibits the ability of DCs to process and/or present antigen [167, 195], tacrolimus immunosuppression has been shown to prolong cardiac allograft survival after *in vivo *injection of mature donor DCs in a transplant model [194]. Before the initiation of a clinical trial, it is therefore important to assess graft survival with each immunosuppressive drug to be used in the trial, with and without DC therapy, in a transplant model.

Another aspect that affects immunogenicity and survival of injected DCs *in vivo* is the route of administration. In one study [242], dexamethasone/LPS-treated DCs prolonged mouse cardiac allograft survival when delivered intravenously, whereas they were ineffective when delivered subcutaneously. Recent evidence in monkeys suggests that autologous tolerogenic DCs may prime an immune response when delivered intradermally, but not when delivered intravenously [39]. In another study in monkeys, intravenous injection of tolerogenic DCs was well tolerated [162].

An unresolved issue in DC therapy for transplantation is the choice between donor and recipient DCs. Most studies have utilized donor DCs or recipient DCs loaded with donor peptides. The OneStudy trial in kidney transplantation has been designed to evaluate autologous DCs not pulsed with donor antigens [39]. The investigators pointed out that a risk of donor sensitization due to the presence of a slight contaminant cell product or the destruction of the injected cells by nonself-recognition [243] could not be excluded using tolerogenic DCs from the donor [39], whereas such a risk should be minimized using autologous DCs. In addition, donor DCs or recipient DCs pulsed with donor peptides require LPS or cytokine stimulation for efficient lymphoid homing and antigen presentation in transplant models (“alternatively activated” DCs) [155, 156]; however, such a stimulation can foster DC maturation. On the contrary, autologous immature DCs do not need LPS stimulation in rodent transplant models [96]. Also, recent evidence suggests donor DCs may die quickly after *in vivo* injection, and the effect of DC therapy may be mediated by endogenous DCs [33, 34], as discussed above. To the opposite, autologous immature DCs have been detected in the spleen of recipient rats at least two weeks after *in vivo* injection [96]. Thus, autologous DCs may offer several advantages over donor DCs as a source for tolerogenic DCs [39]; however, this question has not been answered definitively.

## 6. Concluding Remarks

Studies in rodent and monkey transplant models have provided proof of principle for tolerogenic DC therapy for solid-organ transplantation. Clinical trials testing this strategy have been carried out in patients with type-1 diabetes or rheumatoid arthritis, and a trial in renal transplant recipients has been designed. Nevertheless, several important questions remain. Are autologous DCs superior to donor DCs? Should autologous DCs be pulsed with donor antigen? Are genetically engineered DCs superior to pharmacologically conditioned DCs? Does maintenance drug-based immunosuppression influence the outcome of DC therapy? Which immunosuppressive drugs are most suitable in this regard? What is the best time for DC administration? How can DCs be made truly maturation resistant *in vivo*? As conventional DCs of the recipient seem to be critical for the success of tolerogenic DC therapy, would it be possible to promote tolerance without any cell injection? Answering these questions will likely advance the field and optimize these approaches. Meanwhile, a successful completion of the first clinical trial of DC therapy in transplantation may open a new avenue in the field.

## Figures and Tables

**Table 1:** Recent advances in DC biology, tolerogenic DCs, and pharmacological conditioning protocols.

	Authors	Reference
*Tolerogenic DCs *		
Constitutive ablation of DCs breaks self-tolerance of CD4^+^ T cells leading to fatal autoimmunity	Ohnmacht et al.	[19]
Tolerogenic DCs favor graft tolerance through interferon-*γ* and Epstein-Barr virus-induced gene 3	Hill et al.	[20]
Tolerogenic DCs generated with immunosuppressive cytokines induce antigen-specific anergy and regulatory properties in memory CD4^+^ T cells	Torres-Aguilar et al.	[21]

*DC genealogy *		
DC and monocyte lineages originate from a common progenitor that gives rise to monocytes and committed DC progenitors, which give rise to lymphoid tissue DCs and nonlymphoid tissue DCs	Liu and Nussenzweig	[22]
DCs in mouse lymphoid organs in the steady state are monocyte independent and require Flt3L for their development. Other tissue may contain additional M-CSF-dependent monocytes	Steinman and Idoyaga	[23]
The differing origins of gut DCs may explain how the intestinal immune system manages to destroy harmful pathogens while tolerating beneficial bacteria	Laffont and Powrie	[24]
Comparative genomics reveals functional equivalences between human and mouse DC subsets	Crozat et al.	[25]

*Plasmacytoid DCs (pDCs) *		
Compared to conventional DCs, pDCs show reduced costimulatory molecule expression and poor T-cell allostimulatory capacity. Under homeostatic conditions, nonlymphoid tissue-resident pDCs regulate mucosal immunity and the development of both central and peripheral tolerance	Rogers et al.	[26]
Human pDCs preferentially express immunoglobulin-like transcript 7 (ILT7), which activates an immunoreceptor tyrosine-based activation motif- (ITAM-) mediated signaling pathway	Cao and Bover	[27]

*Pharmacological DC conditioning *		
Rapamycin-conditioned, alloantigen-pulsed DCs present donor MHC class I-peptide via the semidirect pathway and inhibit survival of antigen-specific CD8^(+)^ T cells	Thomson et al. and Fischer et al.	[28, 29]
Human rapamycin-treated DCs are only partially maturation resistant *in vivo *	Macedo et al.	[30]
Adenosine A_2_AR agonist-conditioned DCs attenuate acute renal ischemia-reperfusion injury	Li et al.	[31]
Vitamin D_3_-conditioned DCs induce effector T-cell apoptosis and antigen-specific Tregs	Nikolic and Roep	[32]

*Role of conventional DCs of the recipient in tolerogenic DC therapy *		
Depleting recipient DCs at the time of tolerogenic DC therapy abrogates its beneficial effect	Divito et al., Wang et al.	[33, 34]

*Role of exosomes *		
Exosomes mediate transfer of functional microRNAs between mouse DCs	Montecalvo et al.	[35]
Exosomes from immature DCs plus rapamycin induce tolerance to mouse cardiac allografts	Li et al.	[36]

*Clinical studies of tolerogenic DCs *		
Phase-1 trial of autologous tolerogenic DC therapy in patients with type-1 diabetes	Giannoukakis et al.	[37]
Clinical trials of tolerogenic DC therapy in patients with rheumatoid arthritis	Hilkens and Isaacs	[38]
OneStudy phase-1 trial of autologous tolerogenic DC therapy after kidney transplantation	Moreau et al.	[39]
